# Supplementary material for: Acetaldehyde Enhances Alcohol Sensitivity and Protects against Alcoholism: Evidence from Alcohol Metabolism in Subjects with Variant ALDH2*2 Gene Allele
Source: Biomolecules. 2021 Aug 10;11(8):1183. doi: 10.3390/biom11081183 (PMC8391449; doi:10.3390/biom11081183)
Supplement: Supplementary file 1 [file biomolecules-11-01183-s001.zip › biomolecules-1283763-supplementary.pdf]

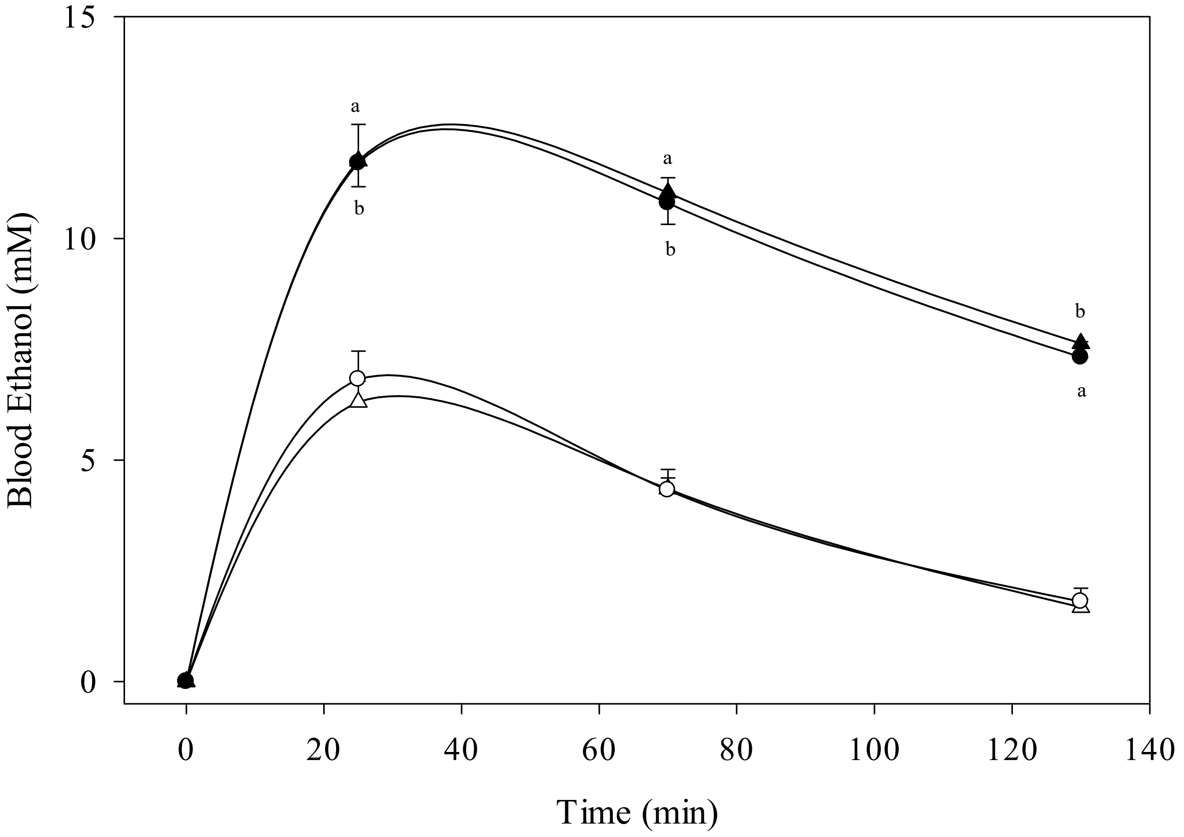

Supplement Figure S1. Blood ethanol concentrations of nonalcoholic subjects with different *ADH1B* allelotypes following different doses of ethanol (0.3 g/kg body weight or 0.5 g/kg). All subjects are *ALDH2*1*1* homozygotes. The study groups are denoted as △, *ADH1B*2/*2* with 0.3 g/kg ethanol (n = 23); ▲, *ADH1B*2/*2* with 0.5 g/kg ethanol (n = 21); ○, *ADH1B*1/*1* with 0.3 g/kg ethanol (n = 10); ●, *ADH1B*1/*1* with 0.5 g/kg ethanol (n = 10). Vertical bars (for clarity only the upper or lower portion shown) represent standard errors of the mean. Statistically significant differences between groups at the corresponding time points was evaluated by ANOVA.

[a]$P < 0.001$ vs. *ADH1B*1/*1* with 0.3 g/kg ethanol.

[b]$P < 0.001$ vs. *ADH1B*2/*2* with 0.3 g/kg ethanol.

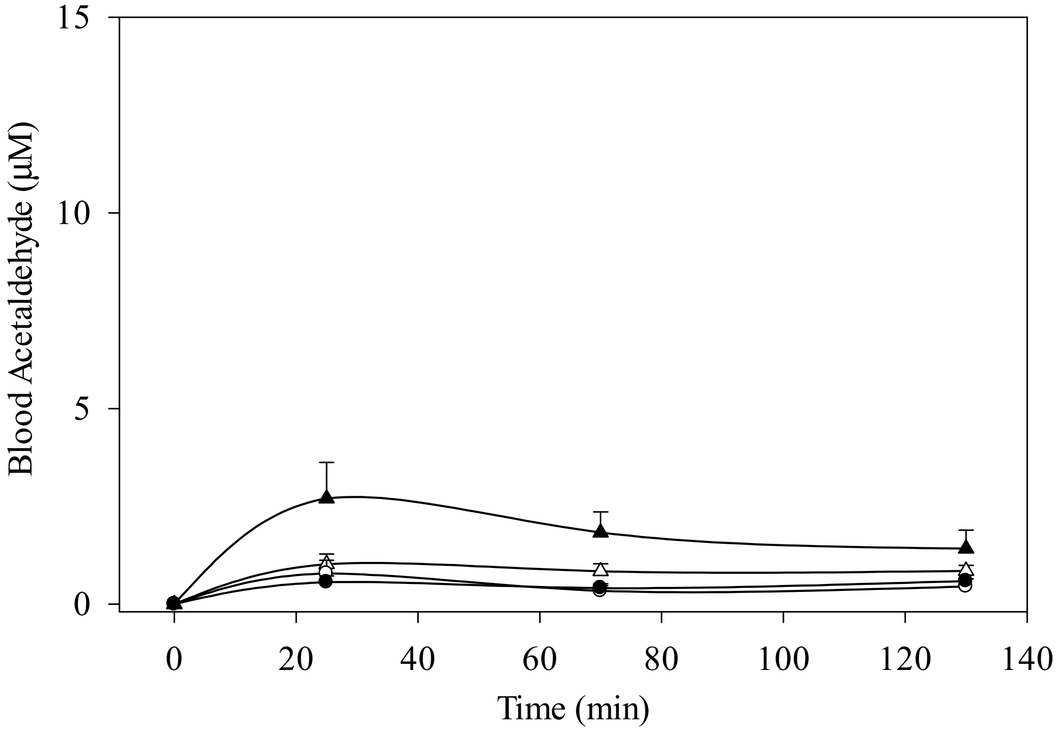

Supplement Figure S2. Blood acetaldehyde concentrations of nonalcoholic subjects with different *ADH1B* allelotypes following different doses of ethanol (0.3 g/kg body weight or 0.5 g/kg). All subjects are *ALDH2*1*1* homozygote. The study groups are denoted as △, *ADH1B*2/*2* with 0.3 g/kg ethanol (n = 23); ▲, *ADH1B*2/*2* with 0.5 g/kg ethanol (n = 21); ○, *ADH1B*1/*1* with 0.3 g/kg ethanol (n = 10); ●, *ADH1B*1/*1* with 0.5 g/kg ethanol (n = 10). Vertical bars (for clarity only the upper or lower portion shown) represent standard errors of the mean. Statistically significant differences between groups at the corresponding time points was evaluated by ANOVA.

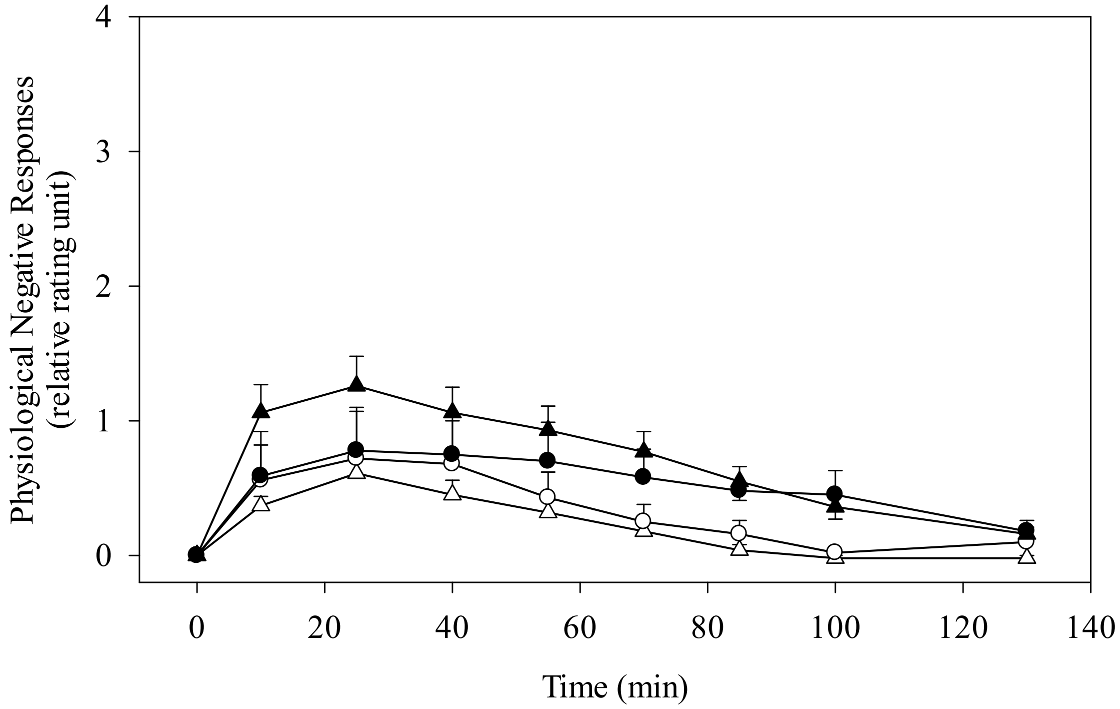

Supplement Figure S3. Alterations in factor of the subjective perceptions of physiological negative responses of nonalcoholic subjects with different *ADH1B* allelotypes following different doses of ethanol (0.3 g/kg body weight or 0.5 g/kg). All subjects are *ALDH2*1*1* homozygote. The study groups are denoted as △, *ADH1B*2/*2* with 0.3 g/kg ethanol (n = 23); ▲, *ADH1B*2/*2* with 0.5 g/kg ethanol (n = 21); ○, *ADH1B*1/*1* with 0.3 g/kg ethanol (n = 10); ●, *ADH1B*1/*1* with 0.5 g/kg ethanol (n = 10). Vertical bars (for clarity only the upper or lower portion shown) represent standard errors of the mean. Statistically significant differences between groups at the corresponding time points was evaluated by ANOVA.

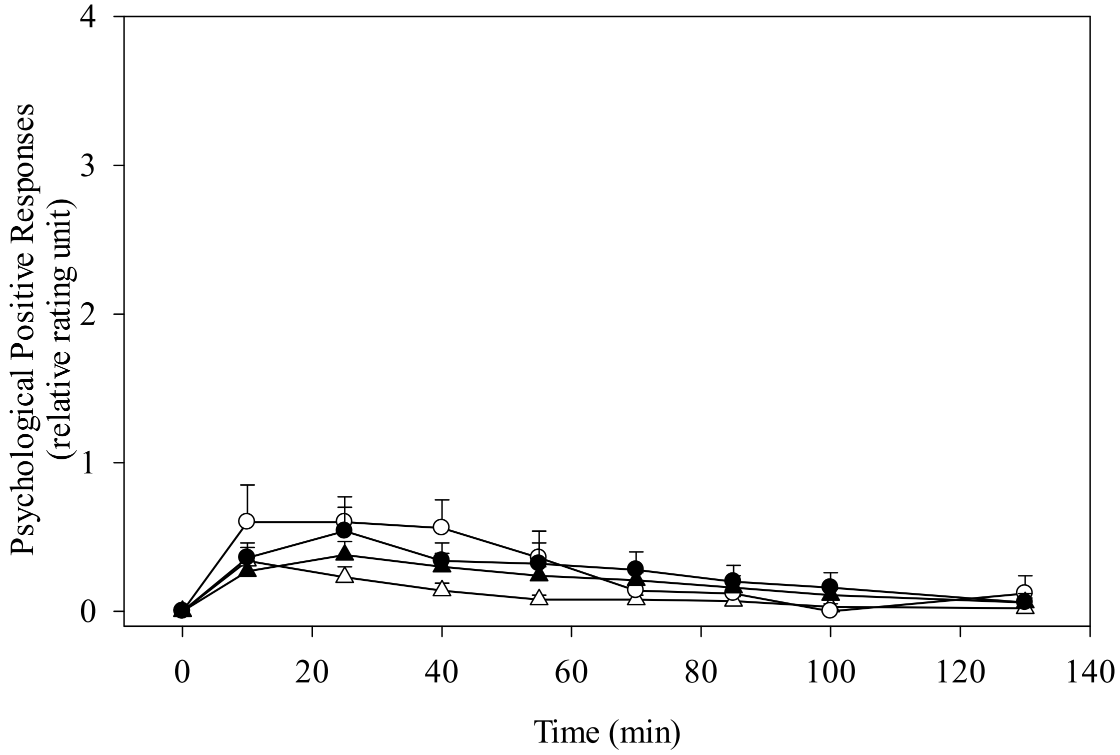

Supplement Figure S4. Alterations of the subjective psychological positive responses in nonalcoholic subjects with different *ADH1B* allelotypes following different doses of ethanol (0.3 g/kg body weight or 0.5 g/kg). All subjects are *ALDH2*1*1* homozygote. The study groups are denoted as △, *ADH1B*2/*2* with 0.3 g/kg ethanol (*n* = 23); ▲, *ADH1B*2/*2* with 0.5 g/kg ethanol (*n* = 21); ○, *ADH1B*1/*1* with 0.3 g/kg ethanol (*n* = 10); ●, *ADH1B*1/*1* with 0.5 g/kg ethanol (*n* = 10). Vertical bars (for clarity only the upper or lower portion shown) represent standard errors of the mean. Statistically significant differences between groups at the corresponding time points was evaluated by ANOVA.
